# The quagmire of alcohol normalization in Nepal: urgent call for a unified alcohol control act

**DOI:** 10.1093/inthealth/ihag021

**Published:** 2026-02-20

**Authors:** Nilam Adhikari, Gampo Dorji

**Affiliations:** School of Public Health and Community Medicine, BP Koirala Institute of Health Sciences, Dharan, Nepal; World Health Organization Country Office for Nepal, Kathmandu, Nepal

## Abstract

Alcohol consumption in Nepal is increasingly normalized despite its substantial health harms. This commentary critically examines how Nepal’s fragmented regulation, weak enforcement, expanding unrecorded alcohol use, and growing commercial influence have undermined effective control. Drawing on national evidence, behavioral theory, and international experience, it argues that reduction requires enforcement mechanisms integrated with sustained behavior-change interventions, rather than regulation alone. Nepal therefore urgently requires a unified alcohol control bill aligned with country’s federal and sociocultural context.

World Health Organization (WHO) identifies ethanol content in alcoholic beverages as a psychoactive and toxic substance with dependence-producing properties, and affirms that no level of alcohol consumption is considered safe for health.^1^ The WHO Global Status Report on Alcohol and Health and the Treatment of Substance Use Disorders (2024) estimates that 400 million people (7% of the global population) aged 15 y and older live with alcohol-use disorders, and an estimated 209 million (3.7% of the adult world population) live with alcohol dependence.^2^ Alcohol consumption is a major risk factor for cancer, liver cirrhosis, non-communicable diseases (NCDs) and mental health disorders.^3^

In Nepal, alcohol consumption is rising steadily. The per capita consumption (APC), a key indicator for population-level alcohol use, grew from 1.4 to 1.5 liters by 2025 and is projected to reach 1.6 liters by 2030.^2^ However, this likely underestimates true consumption due to the widespread use of unrecorded alcohol. National surveys reveal alarming trends; between 2013 and 2022, alcohol consumption increased by 50% in men and 54.9% in women.^4,5^

Alcohol has long been embedded within Nepal’s multifaceted cultural and social traditions, reflecting the country’s ethnic diversity rather than uniform acceptance or rejection. Historically, under the *Muluki Ain* (*old legal code of Nepal*), Nepal’s population was stratified into different categories in which *Tagadhari* (Brahmins and Chhetris), who abstain from alcohol, and *Matawalis* (Specially, Indigenous Nationalities), who can incorporate alcohol into key life events such as weddings, child birth, funerals and festivals. Consumption was largely ritualized and context-bound, not intended for daily use.^6^ Over time, these practices have evolved. Alcohol consumption has transcended ethnic boundaries and become normalized in everyday social interactions and major life events across communities. Once confined to specific groups, alcohol is now widely valorized as a symbol of hospitality, modernity and social status. This shift reflects interacting determinants, including economic liberalization, private investment, alcohol’s contribution to government revenue, aggressive marketing, rapid urbanization and a growing youth population.

Reliable national data on total alcohol production and sales remain unavailable. Nevertheless, the alcohol industry thrives, fueled by rising household incomes through remittances (NPR 1445.32 billion in FY 2023/24). Annual Household Survey (2015–2016) shows that, Nepali households spend NPR 12586 annually on alcohol and tobacco products. Customs records show that imported alcohol alone cost more than NPR 4.81 billion (US$ 34.7 million) in FY 2023/24, highlighting the growing commercial footprint of alcohol in Nepal.

Nepal has committed to reduce the alcohol harm through the Multisectoral Action Plan for NCDs (2021–2025) and implement the WHO’s SAFER initiative. However, alcohol regulation remains fragmented across more than a dozen national laws, including the Liquor Act (1974), Public Health Service Act (2018) and Motor Vehicles and Transport Management Act (1993). These laws address compulsory licensing for production and sales, prohibiting sales to minors (below 18 y), advertising bans and zero tolerance on drink-driving enforcement. However, implementation is inconsistent, regulatory responsibilities are unclear and the existing legal framework is poorly aligned with Nepal’s federal governance structure, limiting their effectiveness.

The national survey reported that 66.3% of drinkers consumed unrecorded alcohol, predominantly traditional homebrews like *aila/raksi* (distilled) (57.4%) and *jaad* (undistilled) (36.7%).^5^ While widely consumed due to their low cost and cultural acceptability, particularly among lower-income populations. However, they pose substantial health risks due to irregular labeling, unknown ethanol percentage by volume and the potential presence of toxic contaminants. Recent government proposals to formally brand or commercialize homemade alcohol risk further increasing its availability, affordability and social legitimacy, thereby exacerbating harm.

Under the Liquor Act, individuals are permitted to produce up to 5 liters of *raksi* and 10 liters of *jaad* annually for personal and cultural use, with production beyond this threshold limit must be reported to tax authorities. In practice, enforcement of these limits is minimal. Although the legal drinking age of 18 y applies to on-premise consumption, explicit regulation of off-premise and online alcohol sales remains absent, despite rapidly expanding digital commerce.

Advertising restrictions remain riddled with loopholes. While Nepal’s Supreme Court upheld a nationwide alcohol advertising ban in February 2023, companies continue to exploit loopholes through surrogate branding, cross-border promotions, and digital marketing.

Evidence from Nepal indicates that reductions in the number of cases of driving under the influence of alcohol or drink-driving-related harm are more likely when behavior-change strategies complement, rather than solely rely on the enforcement. The ‘No Drinking and Driving’ initiative in Kathmandu, which paired sobriety checks with a mandatory 1-h road-safety education session, was associated with a 23% decline in road traffic crashes and a 21% reduction in the injury-to-fatality ratio within one year.^7^ From a Social Cognitive Theory perspective, such gains are plausibly mediated through enhanced self-efficacy, observational learning and self-regulation, suggesting that education-led, norm-shaping interventions can sustain behavior change even in low-enforcement settings.^8^ Such education-led, norm-shaping approaches can sustain behavior change even where continuous policing is neither feasible nor ethically desirable. By contrast, evidence from low- and middle-income countries such as Vietnam and China, where beer has been heavily marketed and has witnessed an increase in consumption, this has not been at the expense of the higher-potency beverages available (World Health Organization 2020).^9^

Nepal’s tobacco control efforts demonstrate what is achievable. The Tobacco Product Control and Regulation Act (2011) provided a unified legal framework and earned the country the WHO Director-General’s Special Recognition Award in 2025. A similar approach is needed for alcohol control.

We believe that, in order to ensure a coherent and effective regulatory framework for alcohol control in Nepal, the Ministry of Health and Population (MoHP), in collaboration with relevant line ministries and key stakeholders, should lead the drafting of a unified alcohol control bill.

This process will likely face significant resistance from industry lobbying, which frames alcohol as economically indispensable, contributing 54.2% of excise revenue and employment. However, global evidence shows that increased alcohol taxation reduces harmful use while increasing public revenue that can be reinvested in health and social programs.^10^

Nepal stands at a critical crossroads. As alcohol use becomes increasingly normalized across social contexts, prioritizing public health over commercial interests is imperative. A unified alcohol control act aligned with WHO’s Global Alcohol Action Plan (2022–2030), SAFER initiative and the country’s contextual context offers an evidence-based pathway to curb alcohol-related harm, close regulatory gaps and integrate enforcement with behavior-change interventions to protect future generations.

## Data Availability

The data underlying this article will be shared on reasonable request to the corresponding author.
